# New molecular targets in acute leukemias: cytoskeletal regulatory proteins

**DOI:** 10.1042/BST20253017

**Published:** 2025-09-22

**Authors:** João Agostinho Machado-Neto, Hugo Passos Vicari, Jean Carlos Lipreri da Silva, Keli Lima

**Affiliations:** 1 Department of Pharmacology, Institute of Biomedical Sciences, University of São Paulo, São Paulo, Brazil; 2 Laboratory of Medical Investigation in Pathogenesis and Targeted Therapy in Onco-Immuno-Hematology (LIM-31), Department of Internal Medicine, Hematology Division, Faculdade de Medicina, University of São Paulo, São Paulo, Brazil; 3 Cancer Institute of the State of São Paulo, Faculdade de Medicina, University of São Paulo, São Paulo, Brazil

**Keywords:** acute leukemias, cytoskeleton, ezrin, stathmin 1, therapeutic targets

## Abstract

Acute leukemias are hematological malignancies characterized by the uncontrolled proliferation of immature bone marrow cells, disrupting normal hematopoiesis. These diseases, classified into acute lymphoblastic leukemia and acute myeloid leukemia (AML), often result from acquired genetic alterations that drive deregulated cell growth and inhibit differentiation. The cytoskeleton has emerged as a promising therapeutic target due to its pivotal role in cellular processes such as adhesion, motility, and division. Among its components, stathmin 1 (STMN1) and ezrin (EZR) stand out for their significant involvement in the pathogenesis and progression of acute leukemias. STMN1, a regulator of microtubule dynamics, is associated with chromosomal instability and leukemic cell proliferation, and is frequently overexpressed in these malignancies. Anti-microtubule agents, such as paclitaxel, eribulin, and cyclopenta[b]indole derivatives have demonstrated the ability to inhibit STMN1 by inducing its phosphorylation at regulatory sites, thereby impairing cell viability and promoting apoptosis. EZR, a membrane-actin linker protein, plays a critical role in cell signaling and tumor survival. Its overexpression has been correlated with poor prognosis in AML. Pharmacological inhibitors like NSC305787 have shown efficacy in reducing cell viability, modulating key pathways such as PI3K (phosphatidylinositol-3-kinase)/AKT (AKT serine-threonine protein)/mTOR (mammalian target of rapamycin), and enhancing the activity of standard chemotherapeutics, thereby supporting their potential use in combination therapies. This review aims to explore the roles of STMN1 and EZR in the pathogenesis of acute leukemias, assessing their potential as therapeutic targets. The goal is to synthesize recent evidence to guide the development of more effective inhibitors, focusing on overcoming therapeutic resistance and tailoring treatments to individual profiles.

## Introduction

Acute leukemias are a group of hematologic diseases characterized by the uncontrolled proliferation of immature bone marrow cells. This abnormal growth compromises the normal production of blood cells, leading to anemia, thrombocytopenia, and functional leukopenia. These conditions can be divided into two main types: acute lymphoblastic leukemia (ALL) and acute myeloid leukemia (AML), depending on the cell line affected [1]. The origin of acute leukemias is often associated with acquired genetic mutations, which favor unregulated cell expansion and inhibition of cell differentiation. These alterations include point mutations, chromosomal translocations, and amplifications of genomic segments [2]. In ALL, alterations such as the t(12;21) and t(9;22) translocations play an important role, not only in defining the subtype but also in the prognosis of the disease [3,4]. In the case of AML, genes such as *FLT3*, *NPM1*, and *CEBPA* are commonly mutated and are highly relevant to the progression of the disease, also influencing therapeutic choices [5,6]. The symptoms of acute leukemia are closely linked to bone marrow failure and the invasion of other tissues by leukemic blasts. These signs may include fatigue, fever, bleeding, and increased susceptibility to infections [7]. In ALL, the appearance of hepatosplenomegaly, lymphadenopathy, and bone pain is common, resulting from the extramedullary infiltration of malignant cells [8].

The diagnosis is based on a detailed clinical evaluation, accompanied by hematological and molecular tests. Analysis of the bone marrow and peripheral blood, together with flow cytometry for immunophenotyping, allows the identification of the type of cell affected and the degree of maturation of the leukemic blasts. Genetic and molecular investigations are crucial to determine the subtype of leukemia, the patient’s prognosis, and the most appropriate therapeutic strategies [9,10]. The World Health Organization (WHO) proposes a classification of ALL and AML subtypes based on genetic and clinical characteristics [11,12]. Treatment of acute leukemias mainly involves intensive chemotherapy, often associated with targeted therapies and, in selected cases, allogeneic hematopoietic stem cell transplantation [13]. In ALL, drugs such as the bispecific antibody blinatumomab, tyrosine kinase inhibitors such as imatinib, and modified T-cell therapies (e.g*.* CAR-T [chimeric antigen receptor T-cell]) have shown promising results for certain subgroups [14,15]. In AML, the use of FMS-like tyrosine kinase 3 (FLT3) inhibitors (such as midostaurin), hypomethylating agents, and B-cell leukemia/lymphoma 2 (BCL2) inhibitors (such as venetoclax) has proven useful, especially in patients with specific mutations or in older age groups [16,17]. Despite therapeutic advances, the prognosis of acute leukemias continues to vary greatly. Children with ALL tend to have survival rates greater than 85%, while adults with AML face more significant challenges, especially when they have high-risk mutations or resistance to initial therapy [18–20].

The cytoskeleton is a protein fiber network in eukaryotic cells, composed of microfilaments, microtubules, and intermediate filaments. Microfilaments, approximately 7 nm in diameter and composed of two helical actin filaments, are essential for cellular motility, signal transduction, and interactions with the extracellular environment and neighboring cells [21]. Microtubules, hollow polar structures about 25 nm in diameter made of α- and β-tubulin dimers, are essential for maintaining cell shape, transporting intracellular materials, and forming structures like spindles, centrioles, cilia, flagella, and neural tubes [22]. Intermediate filaments, approximately 10 nm in diameter, are the most stable and complex cytoskeletal components, playing a key role in cell structure, exhibiting tissue-specific types and distributions, and being closely associated with cell differentiation, although they lack polarity and do not support directional molecular transport [23]. In sum, the cytoskeleton plays a critical role in cellular processes such as shape modulation, cell division, migration, motility, adhesion, cytokinesis, and phagocytosis [24].

In oncology, several therapeutic agents achieve their effects by targeting the cytoskeleton, specifically its protein structures. Among these, drugs that act on microtubules stand out for their significant clinical impact. Recent progress has led to the development of various tubulin inhibitors designed to modulate cytoskeletal dynamics [25]. One category of these drugs, including colchicine and vinblastine, disrupts microtubule polymerization, impairing spindle assembly and limiting tumor cell proliferation [26]. Vincristine holds a significant position in the treatment of hematologic malignancies and is widely used in the therapy of ALL [27] and it stands out as one of the few drugs approved for the management of relapsed T-ALL [28]. Another group, which includes docetaxel and paclitaxel, enhances microtubule polymerization. This action arrests cells in mitosis and triggers apoptosis. Since the 1990s, paclitaxel has been widely used in treating breast, cervical, and non-small cell lung cancers due to its unique ability to stabilize microtubules [26,29]. Eribulin, a third example, differs as a non-taxane inhibitor of microtubule dynamics. By suppressing microtubule growth and reducing overall dynamic activity, eribulin demonstrates potent antitumor effects. Derived from the marine natural product halichondrin B, it shows exceptional preclinical activity and holds promise as an effective anticancer agent [30,31].

Considering the importance of the cytoskeleton in both healthy and neoplastic cells, the lack of specific targeting of drugs that act on these structures against cancer cells can be devastating and cause serious adverse effects. On the other hand, clinically used drugs that act on the cytoskeleton are effective and safe, corroborating the idea that the cytoskeleton is still a valuable target in antineoplastic therapy [32,33]. In this context, even drugs that have a favorable therapeutic window and good efficacy present relevant rates of refractoriness and/or relapse [32–34], indicating the need to identify new targets or drugs with a mechanism of action different from current cytoskeleton-targeting drugs. In this review, evidence on the involvement of stathmin 1 (STMN1), a key protein in the regulation of microtubule dynamics, and ezrin (EZR), the plasma membrane-microfilament linker, in the pathogenesis, progression, and response to therapy in acute leukemias is presented.

## Targeting STMN1 in acute leukemia

STMN1 is an 18 kDa cytoplasmic phosphoprotein also known as oncoprotein 18 (OP18), leukemia-associated phosphoprotein p18 (LAP18), or metablastin. Members of the STMN1 family share a functional domain called ‘stathmin-like,’ which contains up to four phosphorylation sites on serine residues (16, 25, 38, and 68) in the N-terminal region, as well as a specific tubulin-binding domain [35,36]. Its primary function involves destabilizing microtubules by promoting their disassembly (a process known as ‘catastrophe’) or sequestering α/β tubulin heterodimers, thereby preventing microtubule formation [37,38]. Two phosphorylation sites, serines 16 and 63, play a crucial regulatory role; when phosphorylated, they reduce STMN1’s affinity for tubulin heterodimers, constituting a central mechanism of its functional regulation [39].

The regulation of microtubule dynamics is essential for proper cell cycle progression, particularly during mitosis. Various signaling pathways, including MAPK (mitogen-activated protein kinase), PI3K (phosphatidylinositol-3-kinase), CDK (cyclin-dependent kinase), and CamK, target the phosphorylation sites of STMN1 to regulate its activity [39,40]. Additionally, the activation of the JAK2 (Janus kinase 2)/STAT3 (signal transducer and activator of transcription 3) pathway can inhibit STMN1, increasing microtubule stability [41–43]. Dysregulation of STMN1 affects chromosomal segregation, clonogenicity, motility, and cell survival in both normal and neoplastic cells [39].

During normal hematopoiesis, STMN1 expression decreases as hematopoietic progenitors differentiate, accompanied by a reduction in their proliferative capacity. Ectopic overexpression of STMN1 impairs terminal differentiation and promotes progenitor cell proliferation, as demonstrated in studies of megakaryocytopoiesis and erythropoiesis [44–46]. Studies in Stmn1 knockout mice revealed phenotypes consistent with human hematological disorders, such as megaloblastic anemia and thrombocytosis, further supporting its role in erythropoiesis and megakaryocytopoiesis [47].

STMN1 is frequently overexpressed in hematopoietic cells of patients with hematological malignancies, including AML, ALL, myelodysplastic neoplasms with excess blasts, and primary myelofibrosis [43,48–52]. On the other hand, patients with blood cancer who have low cell proliferation rates, such as multiple myeloma, have STMN1 levels similar to healthy donors [53]. This overexpression may compromise chromosomal segregation accuracy, favoring chromosomal instability, a common feature in hematological malignancies typically associated with poor prognosis. The prognostic role of STMN1 in hematological neoplasms remains a topic of debate. While some studies suggest that STMN1 expression does not affect the prognosis of patients with acute leukemias [48,50,51], other research has observed that high STMN1 expression negatively affects the survival of patients with AML [52]. Beyond differential expression, STMN1 actively contributes to the leukemic phenotype, as its specific inhibition reduces the proliferation and clonogenicity of neoplastic cells [43,48,50]. Since hematological malignancies are highly heterogeneous and characterized by alterations in multiple signaling pathways, making the development of effective therapies challenging. In this context, STMN1 integrates several of these pathways, many of which are deregulated in malignant hematopoietic cells, highlighting this protein as a promising therapeutic target in these diseases.

**Figure 1:**
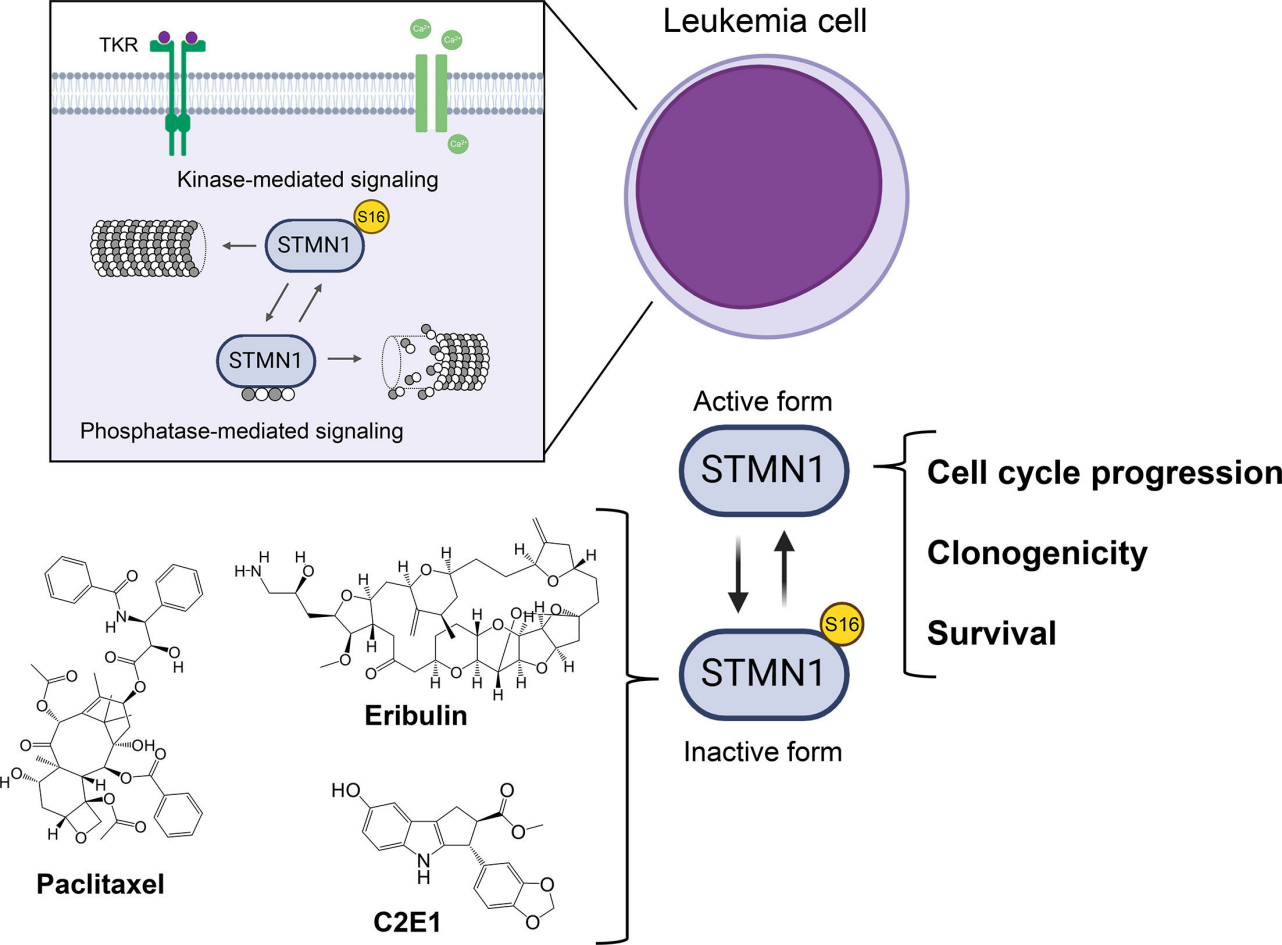
Targeting microtubule dynamics via stathmin 1 (STMN1) modulation in leukemia. Leukemia cells exhibit elevated levels of STMN1 compared with healthy hematopoietic cells, promoting cell cycle progression, clonogenic potential, and enhanced survival. In its non-phosphorylated (active) form, STMN1 induces microtubule catastrophe and sequesters α- and β-tubulin dimers, preventing their polymerization. In its phosphorylated (inactive) form, particularly at serine 16 or 63, STMN1 facilitates microtubule polymerization. Signal transduction pathways mediated by tyrosine kinase receptors (TKR), calcium, and cAMP activate various kinases capable of phosphorylating (**P**) and inhibiting STMN1’s functions (kinase-mediated signaling). Key kinases include cyclin-dependent kinase (CDK), mitogen-activated protein kinase (MAPK), phosphoinositide 3-kinase (PI3K), aurora kinase B (AURKB), protein kinase A (PKA), and Ca2+/calmodulin-dependent protein kinases (CamKs). Conversely, phosphatase-mediated signaling, involving enzymes such as protein phosphatase 2A (PP2A), protein phosphatase 2B (PP2B), and phosphoprotein phosphatase 1 (PP1), dephosphorylates STMN1. The dynamic alternation between the phosphorylated and dephosphorylated states of STMN1 regulates microtubule dynamics. Recent studies have shown that drugs targeting microtubule dynamics, such as paclitaxel, eribulin, and C2E1, induce STMN1 phosphorylation at serine 16, thereby inhibiting its function in acute leukemia cell models. The precise mechanisms underlying this effect remain to be fully elucidated.

Given the substantial body of evidence supporting the involvement of STMN1 in oncogenesis, not only in hematological cancers but also in various solid tumors [54,55], selective STMN1 inhibitors have yet to be developed and could pave the way for significant advances in antineoplastic therapy. In recent years, it has been observed that certain clinically used chemotherapeutic agents, such as paclitaxel and eribulin, both anti-microtubule agents, induce phosphorylation at the inhibitory site (serine 16) and reduce STMN1 expression [43,51,56–58]. Furthermore, a novel synthetic microtubule inhibitor, C2E1, a cyclopenta[b]indole class chemical derivative, was also able to induce STMN1 inhibition through increased phosphorylation at serine 16 [59,60]. These findings have provided insights into the potential expanded use of these drugs in the treatment of hematological neoplasms. In preclinical studies, paclitaxel, eribulin, and C2E1 demonstrated marked antileukemic effects, including the induction of apoptosis and mitotic catastrophe [50,51,57,59,60]. For eribulin, resistance mechanisms have been identified, such as NFκB (nuclear factor-kappa B) activation and efflux pump expression mediated by P-glycoprotein. To address intrinsic resistance, certain pharmacological combinations have been proposed, including the use of elacridar, a third-generation P-glycoprotein inhibitor [57]. A summary of the signaling and cellular processes mediated by STMN1, as well as compounds that target this protein in leukemia cells, is presented in Figure 1.

## Targeting EZR in acute leukemia

EZR belongs to the ERM (ezrin/radixin/moesin) family, that when phosphorylated, its primary role is to connect the actin cytoskeleton to the cell membrane, organizing specialized domains such as apical microvilli [61]. Additionally, EZR serves as a structural support for molecules involved in critical processes, including cell proliferation, migration, adhesion, survival, morphogenesis, and cytoskeletal regulation [62]. Due to these functions, EZR is considered a potential oncogene [63]. Studies have also shown its involvement in signal transduction mediated by tyrosine kinases, contributing to anti-apoptotic signaling pathways [64,65].

EZR consists of three main domains: the FERM (four-point-one/ezrin/radixin/moesin) domain at the N-terminal (~300 residues), a central binding region (~200 residues), and an ERM-associated domain at the C-terminal. The FERM domain, composed of three structural subunits (F1, F2, and F3), forms a compact ‘clover-like’ structure that interacts with integral membrane proteins, structural proteins, Rho regulators (such as Rho-GDI), and phosphatidylinositol-4,5-bisphosphate (PIP2). The highly conserved C-terminal domain plays a crucial function in actin cytoskeleton binding, while the N- and C-terminal domains are connected by a helical structure [66,67]. EZR activation, like other ERM proteins, is dynamically regulated, with Rho positively modulating its activity and RAC potentially exerting inhibitory effects. Phosphorylation of EZR is essential for its function, enabling membrane remodeling and anchoring interactions between the plasma membrane and the actin cytoskeleton [68,69]. Furthermore, EZR overexpression has been linked to the activation of the PI3K/AKT (AKT serine-threonine protein)/mTOR (mammalian target of rapamycin) pathway, which is critical for cell survival and apoptosis evasion. Studies indicate that EZR overexpression increases AKT phosphorylation at serine 473, promoting co-localization with the plasma membrane and reducing apoptosis via mTOR activation [70].

EZR expression varies across tissues and developmental stages. In normal tissues, it is predominantly found in the digestive system, with strong immunoreactivity in epithelial and glandular cells, including intestinal villi. In the nervous system, ezrin expression is detectable in early embryonic stages but diminishes in later development. In the kidneys, epithelial cells of renal acini exhibit strong ezrin staining during human development. Conversely, ezrin expression is absent from endocrine cells, such as thyroid follicular and C-cells, during human development. Recent studies have reported increased ezrin expression in various solid tumors, including breast cancer, melanoma, lung adenocarcinoma, and pancreatic adenocarcinoma, as well as in AML and ALL. This overexpression has been associated with aggressive features, such as enhanced proliferation, migration, and invasion, highlighting its role in tumor progression [67,71–73].

Currently, two synthetic compounds are the best characterized to inhibit EZR function: NSC668394 and NSC305787. These compounds directly bind to EZR, preventing its phosphorylation with micromolar affinity [74]. These inhibitors are valuable tools for studying EZR’s function and hold potential as candidates for therapeutic development in neoplastic diseases [75,76].

In a previous study, the impact of cytoskeleton regulatory genes on clinical outcomes and therapeutic potential in AML was investigated, and EZR was highlighted. High EZR expression was associated with worse prognosis, both in continuous and stratified variable analyses. This association remained significant in multivariate analyses, even after adjusting for age, gender, white blood cell count, and molecular risk, indicating that *EZR* is an independent marker of poor clinical outcomes in AML patients. Additionally, *EZR* mRNA levels were higher in AML patients compared with healthy donors, with no significant association with recurrent mutations [77]. Previously, EZR dysregulation has been linked to the transformation of mouse pre-leukemia erythroblasts into malignant cells [78]. Functional studies have highlighted ezrin as a key mediator in signaling pathways driven by FMS-like tyrosine kinase 3 internal tandem duplication (FLT3-ITD) and mutated c-kit (KIT) receptors, promoting the survival of AML cells [78–80]. Inhibition of EZR function, through the expression of dominant-negative variants, has been shown to reduce proliferation and apoptosis resistance in HS2 leukemic cells [81]. These findings underscore EZR’s role as a key signaling protein in hematopoietic neoplasms.

Functional genomic analyses suggested that EZR contributes to responses to stimuli and regulates key signaling pathways, including PI3K/AKT/mTOR and Rho-GTPases. Pharmacological assays with selective EZR inhibitors, NSC305787 and NSC668394, demonstrated reduced viability, proliferation, and autonomous clonal growth in AML cells. NSC305787 exhibited greater potency, with lower effective concentrations compared with NSC668394. In specific AML cell models, such as MOLM-13 (FLT3-ITD mutated cell line) and Kasumi-1 (KIT^N822K^ mutated cell line), both inhibitors reduced phosphorylation of key proteins (S6RP [S6 ribosomal protein] and 4EBP1 [eukaryotic initiation factor 4E-binding protein 1]) and induced apoptosis, but NSC305787 was more effective in modulating genes related to the cell cycle and apoptosis, including *CCNA2*, *BCL2*, *CDKN1A*, and *BAX* [77]. These findings suggest that high EZR expression is implicated in worse prognosis in AML and that pharmacological inhibitors such as NSC305787 may serve as potential therapeutic agents by inducing cell death and interrupting cell cycle progression.

In ALL, significantly elevated *EZR* mRNA levels were observed, particularly in the B-ALL subtype, correlating with high blast percentages but not survival outcomes or therapy response. These findings suggest EZR’s involvement in ALL pathogenesis rather than treatment resistance. It is interesting to highlight that EZR levels were found to be higher in patients with ALL compared with those with AML. The pharmacological EZR inhibitor reduced ALL cell viability in a dose-dependent manner. It also induces apoptosis, decreases clonal growth, inhibits cell cycle progression, and reduces adhesive and invasive properties of ALL cells [82]. Proteomic analysis highlights its broad impact on biological processes such as RNA catabolism, translation, and mitochondrial function. NSC305787 also modulates PI3K/AKT/mTOR signaling and promotes a tumor-suppressive molecular network, including cell cycle regulators and pro-apoptotic proteins [82].

**Figure 2:**
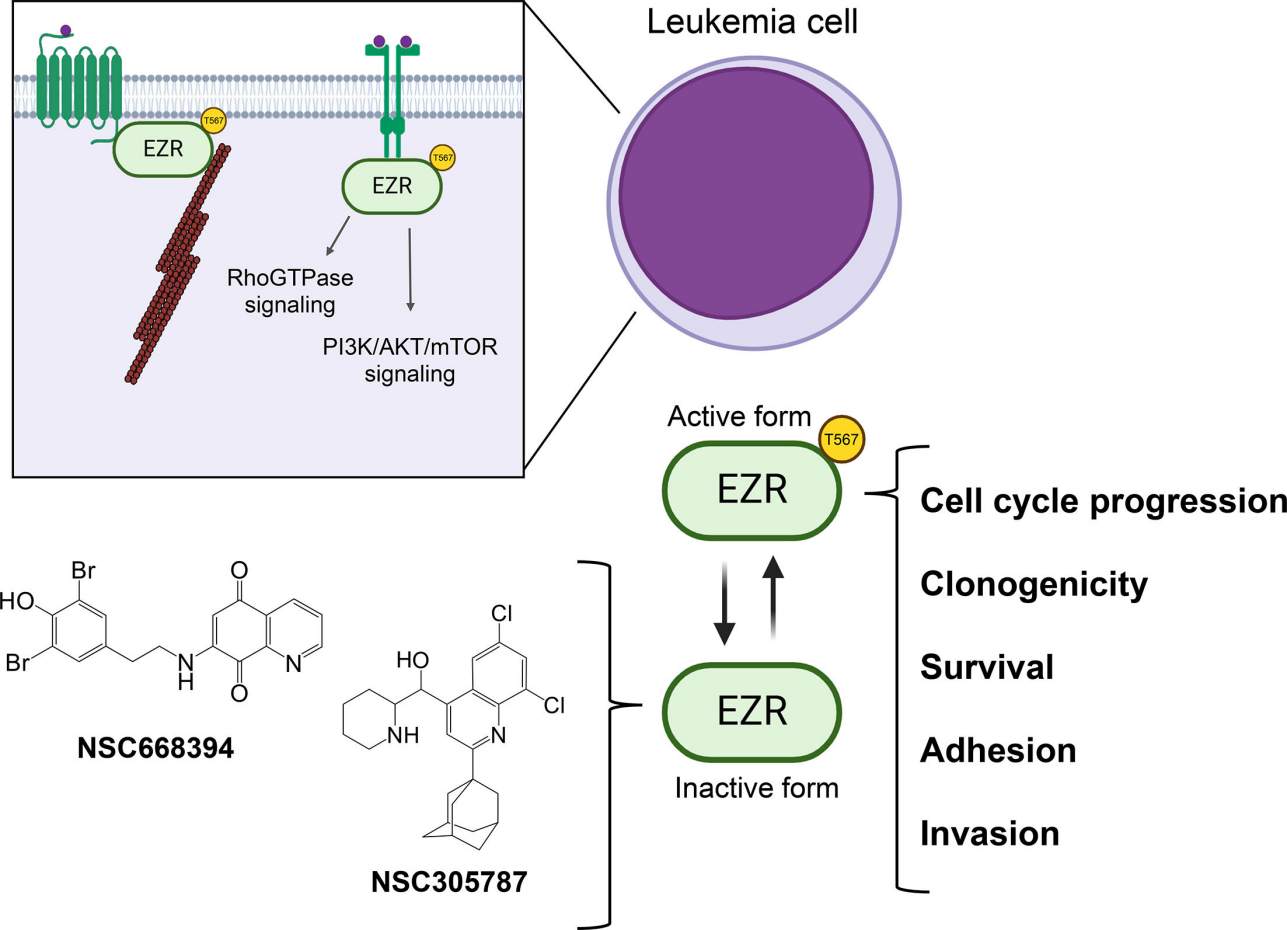
Targeting ezrin (EZR) signaling to inhibit leukemic cell growth and invasiveness. In leukemia cells, EZR expression is significantly elevated, driving cell cycle progression, clonogenic potential, survival, adhesion, and invasion. In its phosphorylated (**P**) form, EZR associates with membrane proteins, including transmembrane receptors, facilitating the linkage between F-actin (actin filaments) and the cell membrane, thereby promoting cytoskeletal reorganization. EZR also interacts with a variety of transmembrane receptors, such as growth factor and cytokine receptors, activating multiple downstream signaling pathways. Key pathways activated by EZR include the PI3K/AKT/mTOR pathway, which supports protein synthesis and cell proliferation, and the Rho GTPase pathway, which regulates migration, invasion, and actin polymerization. Pharmacological inhibitors targeting EZR, such as NSC305787 and NSC668394, have been developed. These inhibitors bind directly to EZR, blocking its phosphorylation and thereby impairing its function and downstream effects.

In addition, NSC305787 enhances the effects of clinically relevant ALL drugs, particularly vincristine, daunorubicin, and cytarabine, showing no antagonistic interactions. In BCR::ABL1^+^ (breakpoint cluster region:: abelson tyrosine kinase 1) ALL, pharmacological EZR inhibition synergizes strongly with dasatinib and imatinib, demonstrating potential as a combination therapy. NSC305787 also effectively reduces viability in primary ALL and AML cells, with greater efficacy observed in ALL that may be due to higher EZR expression. Sensitivity to the inhibitor is independent of risk stratification or recurrent mutations, suggesting its therapeutic potential across diverse patient groups. The selectivity index indicates favorable safety in both acute leukemia cell lines and primary cells, particularly in ALL models [82]. Signaling pathways and cellular processes mediated by EZR, as well as compounds that target this protein in leukemia cells, are summarized in Figure 2.

Previous studies on lymphoid neoplasms have demonstrated that EZR is highly expressed in diffuse large B-cell lymphoma and chronic lymphocytic leukemia. It plays an essential role in organizing the B-cell antigen receptor at the cell membrane, as well as in supporting cell proliferation and survival. In cellular models of these diseases, pharmacological inhibition of EZR was also shown to significantly reduce the malignant phenotype [83,84].

**Perspectives**

- Cytoskeleton plays a fundamental role in cellular processes critical to both normal physiology and cancer biology, making it a promising therapeutic target in hematological malignancies such as acute leukemias. Advances in understanding its components, such as STMN1 and EZR, highlight their involvement in leukemogenesis and potential as treatment targets. Acute leukemias, characterized by uncontrolled proliferation of immature hematopoietic cells, remain challenging to treat despite advances in molecular characterization and targeted therapies. Cytoskeletal proteins like STMN1 and EZR not only regulate essential cellular processes but also contribute to tumor progression, underscoring the value of targeting them for novel therapeutic approaches.
- STMN1, a regulator of microtubule dynamics, is overexpressed in hematological malignancies, promoting chromosomal instability and leukemic cell proliferation. Inhibitors targeting STMN1-related pathways, including anti-microtubule agents like paclitaxel and eribulin, show potential antileukemic effects. Similarly, EZR, a membrane-actin linker, facilitates cell signaling and survival in AML and ALL. Its overexpression correlates with poor prognosis, and pharmacological inhibitors such as NSC305787 have demonstrated efficacy in reducing cell viability and modulating apoptotic pathways.
- Future research should prioritize the development of selective inhibitors targeting STMN1 and EZR, with a focus on improving their potency and specificity. Combining these inhibitors with existing chemotherapeutics or targeted therapies, such as FLT3 (FMS-like tyrosine kinase 3) or BCR::ABL1 (breakpoint cluster region:: abelson tyrosine kinase 1) inhibitors, holds significant potential to improve outcomes, particularly for high-risk patients. Moreover, elucidating resistance mechanisms and tailoring therapies to individual cytoskeletal profiles will be crucial to overcoming therapy refractoriness and reducing relapse rates, paving the way for more effective and personalized treatment strategies.

## Open Access

This article has been published open access under our Subscribe to Open programme, made possible through the support of our subscribing institutions, learn more here: https://portlandpress.com/pages/open_access_options_and_prices#conditional

## Abbreviations

AKT: AKT serine-threonine protein
ALL: acute lymphoblastic leukemia
AML: acute myeloid leukemia
BAX: Bcl-2-associated X protein
BCL2: B-cell leukemia/lymphoma 2
BCR::ABL1: breakpoint cluster region:: abelson tyrosine kinase 1
CAR-T: chimeric antigen receptor T-cell
CCNA2: cyclin A2
CDK: cyclin-dependent kinase
CDKN1A: cyclin-dependent kinase inhibitor 1A
CEBPA: CCAAT enhancer binding protein α
4EBP1: eukaryotic initiation factor 4E-binding protein 1
ERM: ezrin/radixin/moesin
EZR: Ezrin
FERM: four-point-one/ezrin/radixin/moesin
FLT3: FMS-like tyrosine kinase 3
FLT3-ITD: FMS-like tyrosine kinase 3 internal tandem duplication
JAK2: Janus kinase 2
KIT: c-kit receptor
LAP18: leukemia-associated phosphoprotein p18
MAPK: mitogen-activated protein kinase
NFκB: nuclear factor-kappa B
NPM1: nucleophosmin 1
OP18: oncoprotein 18
PI3K: phosphatidylinositol-3-kinase
PIP2: phosphatidylinositol-4,5-bisphosphate
S6RP: S6 ribosomal protein
STAT3: signal transducer and activator of transcription 3
STMN1: stathmin 1
WHO: World Health Organization
mTOR: mammalian target of rapamycin
